# Strychnia Hypodermically in Malarial Hæmaturia

**Published:** 1886-11

**Authors:** S. Leard Keown

**Affiliations:** Dallas, Texas


### STRYCHNIA HYPODERMICALLY, IN MALARIAL HÆMATURIA.

*By S. Leard Keown, M. D., Dallas, Texas.*

For Daniel's Texas Medical Journal.

MR. F—, Oct. 26, just four weeks from a malarious district in Arkansas, where he had been having chills for 12 months, causing anæmia, emaciation, enlargement of the liver and spleen, was a very unfit subject to stand the trying ordeal of this dreaded disease.

He had enjoyed an immunity from chills since leaving Arkansas, until the 9th of April, when he had a light chill. This put him on his guard, and on the following day, after taking a course of purgative medicine, took a liberal quantity of quinine; this did not have the desired effect in keeping off the expected chill as it made its return a little earlier than it appeared on the day before. The same course of medicine was repeated, with the hope of preventing another paroxysm, which was as fruitless as in the first instance, as on this, the third day from his first chill, he had one of unusual severity, lasting two and a half hours, during which time he was in hard rigors. This attack was attended with considerable fever and inordinate vomiting. The distress, occasioned by his constant vomiting, caused him to send for me, and I reached him about two o'clock p. m. just as the cold stage was going off. He was still vomiting bilious matter which was expelled in small quantities every few minutes. There was increased action of the salivary glands, with the same discoloration of saliva as was shown by the saliva vomited. Temperature was 104°, circulation 140°, with very low arterial tension. Inquiring into the appearance of his urine, I was told it was very dark; this, with his general symptoms, aroused my suspicions of the existing trouble; however, I refrained from coming to a settled conclusion, till his urine could be inspected.

I gave him calomel, two grains, sub. nit. bismuth, four grains and morphine one-fourth grain. This was borne by the stomach, notwithstanding its extreme irritability. The vomiting continued though nausea and retching was not so severe. A half teaspoonful each, of spirits of nitre and fluid extract of ergot was given which was immediately ejected from the stomach, although it was given largely diluted. A mustard plaster was placed over the stomach, and allowed to burn quite freely; his feet were placed in warm water and the same dose of nitre and ergot repeated, which was as promptly expelled as the first dose. Directing quinine by inunction to be commenced at six o'clock, and trusting to the calomel, bismuth and morphine, which latter was reduced from one-fourth to one-eighth grain, to keep the severe vomiting somewhat restrained, I left, with instructions for them to send for me, if the next urine voided was still dark. They failed to save a specimen of urine, or to even notice it, as he went out of the house after dark to urinate. Fortunately I was called again in the early part of the night on account of the troublesome vomiting which was on the increase, as regards both severity and quantity. My apprehensions of malarial hæmaturia now run so high that I determined to remain with him, till he again passed his urine. In the meantime I engaged myself in addressing remedies to palliate the obstinate vomiting which continued in defiance of every anti-emetic remedy at hand. While other remedies were being tried to quiet the stomach, warm water, as hot as could be borne, was given him. This would so dilute the acrid contents of the stomach as to ward off vomiting for a while. It was also observed that when vomiting did occur immediately after taking the warm water, the act was accomplished with more ease. His thirst was intense, and every spoonful of cold water given him was instantly thrown off. We now abandoned all attempts to check emesis and made constant use of the hot water draughts, whenever nausea appeared. This being retained a short while, gave some relief to his thirst in addition to its service in mitigating the hard vomiting. About two o'clock in the morning he discharged from the bladder a half pint of fluid as dark as venous blood.

I now knew that my former suspicions were correct, and at once commenced to use strychnia hypodermically, giving one thirty-fifth of a grain at a time. The first made an immediate perceptible change in the circulation. The pulsation became quicker and stronger, with the intervals between each pulsation clearly defined. Directing the calomel, bismuth and morphine to be continued every four hours, and the warm water given whenever vomiting was threatened, and a continuance of the quinine by inunction, I left to return again at daylight. On my return I found vomiting unchanged, arterial tension again lowering, and prostration rapidly advancing. His skin by the approaching daylight showed to be almost as yellow as an orange. His respiration was becoming hurried, and he was complaining of great bodily heat though his temperature had gone down to 100°. I now injected the second dose of strychnia, with a full dose of morphia. An enema of warm water and soap was given, which caused the bowels to move, the action being watery and very yellow. After giving him a little rest, the syringe was again used, which caused a larger action than the former, and of the same bilious appearance. The bismuth and morphine were left off now, as the former seemed to be of no use, and the latter was not needed. Calomel was continued in five grain doses every four hours for the following twenty-four hours. At this time the color of the urine was not so bad, but its quantity was very scant. Eighteen hours elapsed without any urine being passed, which was a period of great anxiety to me, for on the appearance of this fluid I mainly depended for my prognostic information. As soon as this failure on the part of the kidneys was noticed, I commenced giving five drops of tincture of digitalis with each hypodermic injection of strychnia. When he again passed his urine, to my delight, it had very much improved in appearance. While his temperature had gone to the normal point, skin was rapidly clearing up, vomiting was less frequent, and bowels were discharging bilious matter freely, I felt an extreme uneasiness on account of the kidneys failing to act for this length of time.

Strychnia and digitalis were kept up, but their interval between administration was lengthened from four hours to six hours. By this time (the fourth day since the hard chill) the symptoms had improved, but prostration was considerable, and exhaustion was expected. The stomach was too irritable to retain nourishment of any ordinary kind, or to carry out its part in the digestive process. The bowels were moving too often to allow of rectal feeding. Now that the alarming symptoms had considerably abated, a fatal issue impended from the source of exhaustion. During my absence, some of his friends, in the attempt to nourish him, gave a small quantity of sweet milk which was in a few minutes vomited in a hard coagulated condition. Under these embarrassing circumstances attending the administration of nourishment, the peptonising powder of Fairchild Bros. & Foster was used in the artificial digestion of milk, which was taken with a relish and retained with a joyful surprise by the patient. He was again seen in twenty-four hours, by which time it was evident that recovery would be the result.

The essentials in this clinical report are the heroic use of strychnia, to which allusion was made by myself in DANIEL'S TEXAS MEDICAL JOURNAL, October edition, 1885, and the use of chemically digested nourishment. Strychnia, standing at the head of vaso-motor stimulants, produces contraction of the relaxed blood vessels upon which the leakage depends in malarial hæmaturia, etc.; hypodermic administration is greatly to be preferred in such cases, on account of the extreme irritability of the stomach, and to secure immediate action. Some difficulty exists, however, in making a suitable solution for this mode of administration, unless the following plan, which I obtained by experiment, is used:

Take powdered crystalized sulphate of strychnia one grain, water ten drops, alcohol sixty drops; mix in a test tube, and apply heat till solution is effected. This makes a permanent solution, which is not precipitated by the addition of either alkalies or acids, each drop representing one-seventieth of a grain of the alkaloid. A sufficient quantity of this solution should be used to effect contraction of the blood vessels. We are authorized by Prof. Orendorf, professor of Therapeutics in the Kentucky School of Medicine, to use one-thirtieth, one-twentieth, one-tenth of a grain if necessary, to effect this result. In this case the sub-cutaneous injection of one-thirty-fifth of a grain every four to six hours kept up contraction of the vessels, and arrested the escape of blood and bile from their proper channels. No unpleasant symptoms were produced throughout the whole use of the drug in this case. For a few minutes after injecting the dose used, twitching of the muscles and tendons of the forearms was noticed, but no other threat of convulsive action was made.

It is a fact, conceded by many observing physicians, that not a few patients die of exhaustion after surviving the crisis of the disease. Suitable nourishment at the proper time would save many of this class. The stomach, if not too irritable to retain ordinary nourishment, is too feeble to digest the same; besides, the time required for stomach digestion is too valuable to loose, when life depends on immediate nutrition. Of all the prepared foods tried in my practice, I have not seen the utility of the one here mentioned, surpassed. *Fresh, pure milk,* which contains a greater variety of the nutritive elements required by the system, can be introduced into the stomach pre-digested, in which condition it is at once appropriated by the system.

I am aware that many of our best practitioners place more confidence in heroic doses of quinine, and a free use of jaborandi in the treatment of malarial hæmaturia, than they do in the employment of strychnia. Quinine, when given in large doses, certainly has the effect of paralyzing the vaso-motor centres, and thereby relaxes the blood vessels and bile ducts; besides, when the blood is saturated with quinine, the amœboid movement of its corpuscular elements is arrested, making the remaining portion, its plasma more fluid; in which state it more easily passes through the relaxed tissues of the vessel. Jaborandi is undoubtedly the most relaxing therapeutic agent in use. This erroneous use of these drugs in malarial hæmaturia is, to some extent, pardonable; for this disease is known to depend upon malaria; and quinine is our foremost antidote to this miasm; and the great healing agent to its pathological lesions. The ability of the skin to act vicariously for the kidneys, strongly suggests the employment of jaborandi, which has the power to augment the action of the sudorific glands to their utmost extent. While the use of these agents is thus strongly indicated, we must not fail to remember their relaxing effects upon the whole system, which, to overcome this undue relaxation, is the great desideratum in treating this, the most dreaded of malarious affections. After the unpleasant experience of the responsibilities in a good number of cases of this trouble, we have brought our convictions to the point to abandon the use of jaborandi altogether, and to make a *very careful* use of quinine. Malarial hæmaturia, depending, as it does, upon malarial miasm for its pathological foundation, the importance of removing the *cause* in treating any disease, and of preventing the recurrence of paroxysms in malarial affections, and the necessity of using supportive measures in this disorder, which is attended by early prostration, renders a *moderate* use of quinine imperative. We may give a sufficient amount of the alkaloids of cinchona to partially cinchonize the patient, without producing relaxation of the system. The physiological action of quinine is, in moderate doses, to stimulate all parts of the system; while in large doses it paralyzes the vaso-motor centres. (Farguharson, Bartholow, Chisom, Lewisky, Birquet, et al).

---

DANIEL'S TEXAS MEDICAL JOURNAL is read more extensively in Texas than any journal published, and the circulation is still increasing. Send along your names, gentlemen, there will be enough to go around.

